# The effect of intracortical bone pin on shoulder kinematics during dynamic activities

**DOI:** 10.1080/23335432.2019.1633958

**Published:** 2019-06-30

**Authors:** Maryam Hajizadeh, Benjamin Michaud, Mickael Begon

**Affiliations:** Laboratoire de Simulation et Modélisation du Mouvement, Faculté de médecine, Université de Montréal, Laval, QC, Canada

**Keywords:** Intracortical bone pins, Scapulohumeral rhythm, arm movement, kinematics

## Abstract

Intracortical bone pins are introduced as gold standard for analysing skeletal motion because of eliminating soft tissue artefact. However, excluding this methodological error might be in cost of intervening movement pattern by local anaesthesia and pain of external tool within body. The purpose of this study was to examine whether intracortical bone pins alter shoulder joint kinematics or coordination. Three subjects were analysed during arm elevation/depression in frontal and sagittal planes. Retroreflective skin markers captured the motion in two sessions, before and after inserting bone pins (SKIN and PIN sessions), respectively. Thoracohumeral and scapulothoracic kinematics and scapulohumeral rhythm (SHR) were compared between two sessions. Thoracohumeral exhibited lower elevation and internal rotation in PIN session especially close to maximum arm elevation. The highest differences were observed for scapulothoracic kinematics, with higher retraction during abduction as well as higher posterior tilt, lateral rotation and retraction during flexion in PIN session. In addition, no systematic changes in SHR between subjects was found. Statistically significant lower SHR in PIN session was observed over 87-100% of thoracohumeral elevation/depression cycle in frontal plane and over 25-61% in sagittal plane. Further studies should treat carefully toward the clinical validity of shoulder joint kinematics after inserting bone pins.

## Introduction

Retroreflective markers mounted on either skin or intracortical bone pins are used to highly accurately capture shoulder motion and evaluate the shoulder dynamics (Fayad et al. 2008; Dal Maso et al. 2014). Skin-mounted markers are more commonly used because of their non-invasiveness. However, the soft tissue artefact is an inevitable component of the resulting kinematics (Maiwald et al. 2017). Several numerical and experimental solutions have been suggested to reduce this artefact. Bone-anchored markers have been introduced as an experimental methodology to overcome this major issue. Its invasiveness explains both the limited number of studies and the small sample size. While the direct movement of bones can be benefited for methodological and validation studies (Cereatti et al. 2017), the use of pins is also accompanied with negative aspects for clinical inference: local anesthesia for inserting pins, interference of pins in the motion of muscles, tendons, or ligaments, painful movements with pins and psychological effect of an external tool within body (Maiwald et al. 2017). Maiwald et al. (2017) have previously looked at such effects on foot dynamics. Based on ground reaction force, subjects tended to have less striking foot contacts after inserting pins. They reported that gait pattern does not systematically change by implanting pins. Due to different anatomy and mechanical structures of shoulder complexity, however, it might exhibit dissimilar behaviour compared to the foot.

The effect of bone pins could change the motion of scapulothoracic or glenohumeral joints. Furthermore, the coordination between these joints can be altered as neurological and psychological effect to stimulate similar motion for shoulder complex before and after pin insertion. Scapulohumeral rhythm (SHR) represents any changes in scapulothoracic/scapulohumeral movement and their coordination (Inman and Abbott 1944). While able-bodied subjects exhibited an average ratio of 2:1 for glenohumeral against scapulothoracic motion in 2D static abduction position (Inman and Abbott 1944), a large variability (range of 1.25–7.9) exists between subjects and studies when SHR is calculated as the ratio of glenohumeral elevation to scapulothoracic upward rotation using 3D motion analysis system (Braman et al. 2009; Yoshizaki et al. 2009). The variability results from the fact that these two angles are not coplanar. Moreover, numerical instability may occur for low-arm elevation. Recently, SHR has been calculated as the ratio of the 3D glenohumeral joint contribution against the 3D scapulothoracic joint contribution to the total arm elevation to overcome these issues (Robert-Lachaine et al. 2015). Nevertheless, altered biomechanics or stability associated with shoulder dysfunction affects the SHR (Warner et al. 1992; Kibler et al. 2002). For example, Fayad et al. (2008) showed lower SHR in patients with glenohumeral osteoarthritis or frozen shoulder due to higher scapular lateral rotation. Similarly, patients with rotator cuff tear increased the scapular contribution to arm elevation leading to lower SHR. Such strategy has been considered as a positive adaptation for individuals to reach higher function level (Mell et al. 2005). Our objective was to assess how inserting intracortical bone pin alters thoracohumeral and scapulothoracic kinematics, as well as shoulder joint coordination.

## Methods

Four healthy male subjects volunteered to participate in this study. Experimental protocol is fully described in Dal Maso et al. (2014). The inclusion criteria were no history of pain or dysfunction in the shoulder. All participants showed the ability of generating normal range of motion (ROM) by getting scores lower than 10.5 at the Disabilities of the Arm, Shoulder and Hand questionnaire (Hudak et al. 1996). The participants, called as S1, S2, S3, S4 (32, 27, 41, 44 years old, 172, 165,182, 177 cm, and 80, 57, 82, 115 kg, respectively) signed an informed consent form being approved by the Karolinska Institute (Sweden) and the Université de Montréal (Canada) ethics committees.

Data collection was performed in two sessions in a single day, for each participant. For session 1 (SKIN), 22 skin markers were attached to the left clavicle (5), scapula (4), humerus (7) and thorax (6) based on the model introduced by Jackson et al. (2012). Their locations were previously marked on the skin with a pencil to replace them accurately if they had to be removed during the surgery. For session 2 (PIN), three intercortical bone pins were added (left clavicle, scapular spine and deltoid insertion). The insertion of pins was performed by a surgeon, following local anaesthesia (see Dal Maso et al. (2014) for details). Pin locations were adjusted to avoid muscles, nerves and blood vessels as well as any contact of pins with head, neck and skin markers during movements. Marker trajectories were recorded using 18-camera VICON^TM^ optoelectronic motion analysis system (Oxford Metrics Ltd, Oxford, UK) at 300 Hz.

Anatomical and relaxed static positions were primarily recorded for each person per session. Thereafter, each participant was asked to perform movements in SKIN session and repeat these movements in PIN session. The movements included functional motion tasks and main tasks. Functional motion tasks were arm flexion, rotation, and circumduction performed in each session to find functional joint centres and axes. Main tasks involved 10 trials of arm elevation/depression in frontal plane (abduction/adduction) and sagittal plane (flexion/extension). In S4, the location of the scapula’s pin interfered with the skin markers which had to be repositioned more laterally. The post-surgery skin markers on the scapula were quasi-collinear. This resulted in the impossibility of accurate kinematics calculations. S4 data were removed.

The systems of coordinates and sequences were defined based on International Society of Biomechanics (ISB) recommendations (Wu et al. 2005). For both SKIN and PIN sessions, thoracohumeral and scapulothoracic kinematics as well as SHR according to Robert-Lachaine et al. (2015) algorithm were calculated from the skin-mounted markers for all available trials of arm elevation/depression in frontal and sagittal plane. To calculate SHR it is primarily necessary to extract the contribution of each joint, as total arm elevation is composed of 3D contribution of glenohumeral and scapulothoracic joints. The glenohumeral position is set to its reference posture, and total arm elevation is recomputed to retrieve the scapulothoracic contribution. There, the glenohumeral contribution is obtained by subtracting the primary total arm elevation from the re-computed one (Robert-Lachaine et al. 2015). Peak values for SHR were also reported for each subject, each task and each session, separately. The effect of inserting bone pins on thoracohumeral and scapulothoracic rotations was interpreted based on the observations for each individual. Statistical analyses were performed only for SHR using the common ROM in thoracohumeral elevation for all the subjects: [10° to 108°] in frontal plane and [14° to 100°] in sagittal plane. The SHR was normalized to 50 points for arm elevation (1–50% of the cycle) and 50 points for arm depression (51–100%). Then, non-parametric paired *t*-tests using statistical parametric mapping (SPM) were implemented, during elevation and depression separately, to determine whether any significant differences exist between SKIN and PIN sessions. The open-source SPM1d toolbox (http://www.spm1d.org) in MATLAB (R2018b, Mathworks Inc) was used for our SPM analysis.

## Results

### Descriptive statistics

**Figure 1.**
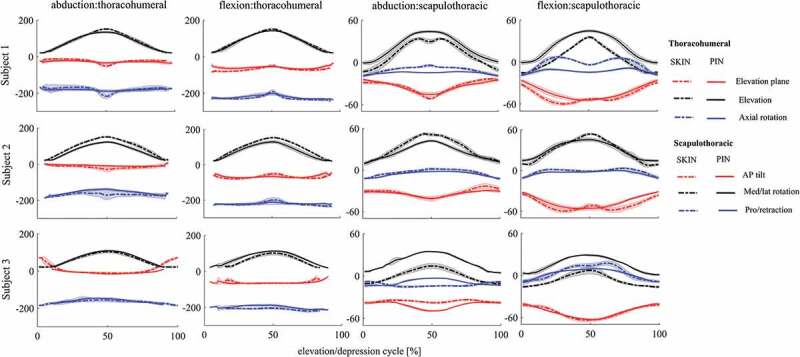
Thoracohumeral and scapulothoracic kinematics during arm elevation/depression in frontal plane (abduction) and sagittal plane (flexion) for each subject. Dotted line shows the SKIN session and filled line shows the PIN session. Thick lines show the mean value and the shaded areas show standard deviation. SKIN/PIN: session before/after bone-pin insertion.

**Figure 2.**
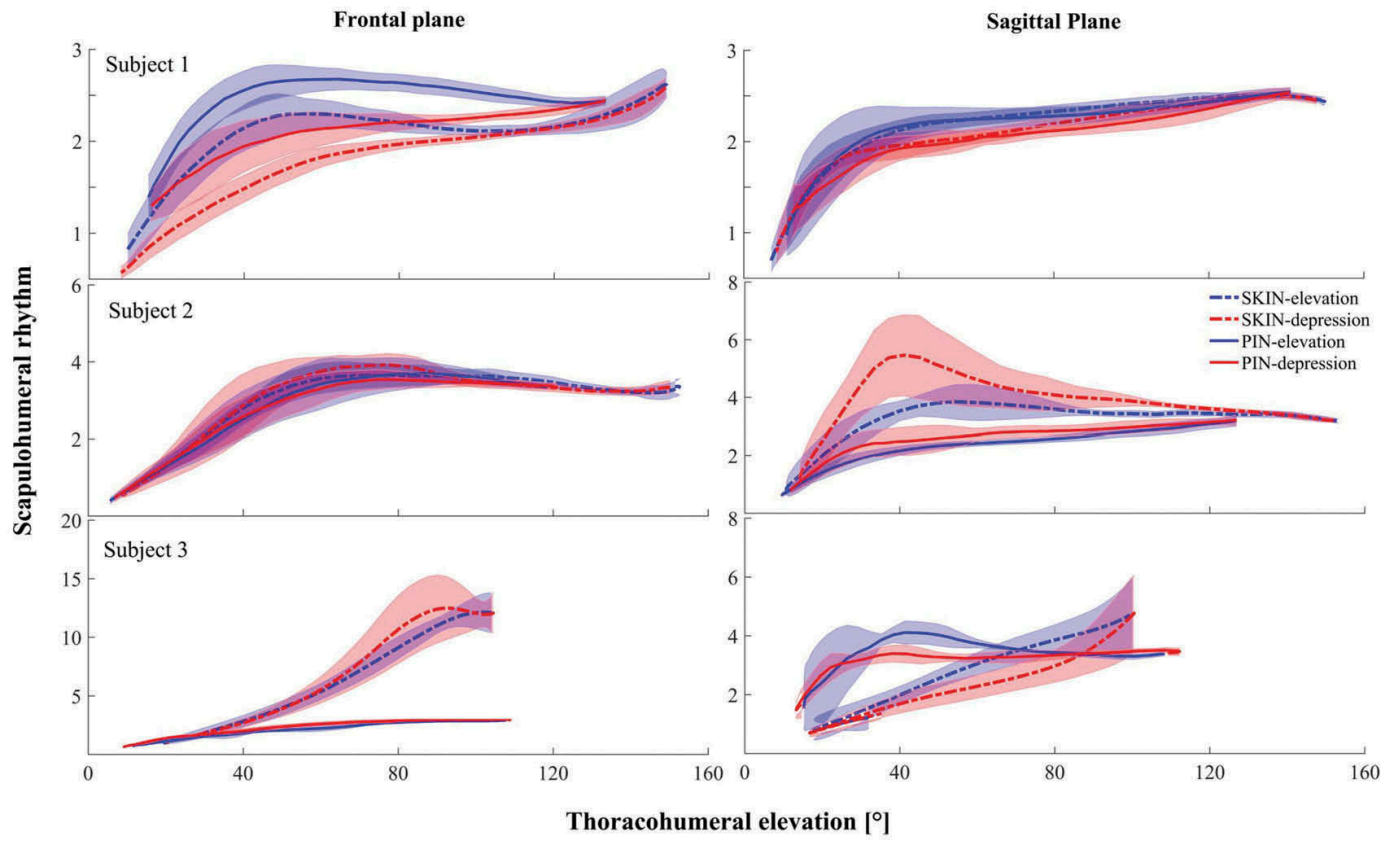
SHR during arm elevation/depression in frontal (left) and sagittal (right) plane. Blue shows elevation, red depression, dotted lines SKIN session and filled lines PIN session. Thick lines show the mean value and the shaded areas show standard deviation. SKIN/PIN: session before/after bone-pin insertion.

*S1*: In *frontal plane*, this subject exhibited similar thoracohumeral kinematics in both sessions except for the interval near maximum elevation. Lower elevation at this interval led to lower RoM for the plane of elevation as well as axial rotation in PIN session (Figure 1, Table 1). Scapulothoracic kinematics showed higher lateral rotation and retraction in PIN session, while the subject produced lower RoM for scapulothoracic in this session. In addition, SHR was higher in PIN session during its whole available range of thoracohumeral elevation compared to SKIN session (Figure 2). The maximum rhythm occurred at the maximum thoracohumeral elevation for SKIN session compared to middle range of thoracohumeral elevation for PIN session during arm elevation, and it was achieved at highest thoracohumeral elevation for both sessions during arm depression (Table 2). In *sagittal plane*, the subject generated similar thoracohumeral kinematics for both sessions, while he generated lower RoM for axial rotation in PIN session. Scapulothoracic kinematics showed higher lateral rotation, retraction and posterior tilting during PIN session. The pin insertion also led to lower RoM for retraction of scapulothoracic joint. SHR showed similar pattern and values for SKIN and PIN sessions. The SHR showed an increase during the whole elevation, where the rate of change decreased after early phase of elevation (Figure 2, Table 2). In frontal and sagittal planes, SHR was higher during elevation compared to depression for both sessions (Figure 1, Table 1).

**Table 1.** Range of motion for thoracohumeral and scapulothoracic rotations during SKIN and PIN sessions.

	Thoracohumeral			Scapulothoracic		
		)Abduction (°	Flexion (°)		Abduction (°)	Flexion (°)
Subject 1	Plane of elevation	39.5 ± 6.6	33.2 ± 6.0	Anterior/posterior tilt	28.4 ± 1.2	30.9 ± 2.6
		19.7 ± 5.8	25.0 ± 5.2		21.4 ± 2.6	29.0 ± 1.5
	Elevation/depression	130.5 ± 1.4	129.6 ± 2.1	Medial/lateral rotation	48.4 ± 2.9	51.9 ± 1.7
		113.9 ± 4.7	121.4 ± 2.4		45.2 ± 3.4	46.4 ± 3.3
	Axial rotation	52.5 ± 9.9	45.8 ± 10.4	Protraction/retraction	14.8 ± 2.1	25.6 ± 1.5
		18.4 ± 8.5	31.2 ± 8.4		6.6 ± 0.9	11.3 ± 1.0
Subject 2	Plane of elevation	33.0 ± 12.9	30.8 ± 3.4	Anterior/posterior tilt	19.1 ± 4.0	26.2 ± 2.4
		13.1 ± 4.0	32.4 ± 8.0		12.0 ± 1.1	25.3 ± 2.7
	Elevation/depression	130.0 ± 2.8	132.8 ± 1.8	Medial/lateral rotation	43.1 ± 1.9	47.1 ± 2.1
		99.0 ± 4.8	110.7 ± 5.8		32.9 ± 3.9	32.3 ± 2.4
	Axial rotation	36.0 ± 16.1	39.0 ± 14.0	Protraction/retraction	14.8 ± 1.5	14.8 ± 1.3
		39.8 ± 5.2	20.2 ± 9.4		10.8 ± 0.5	11.8 ± 1.0
Subject 3	Plane of elevation	16.8 ± 0.2	129.6 ± 6.4	Anterior/posterior tilt	5.7 ± 1.0	18.6 ± 3.8
		14.6 ± 1.7	33.1 ± 5.2		12.9 ± 0.9	23.5 ± 1.6
	Elevation/depression	85.0 ± 5.8	69.4 ± 14.6	Medial/lateral rotation	20.4 ± 0.9	19.7 ± 7.8
		89.5 ± 2.2	92.2 ± 3.0		30.3 ± 0.7	28.3 ± 0.3
	Axial rotation	9.1 ± 1.9	22.3 ± 2.8	Protraction/retraction	5.4 ± 0.4	23.9 ± 3.4
		26.7 ± 0.3	27.7 ± 5.2		11.7 ± 1.0	20.6 ± 2.3

The range of motion is shown for three degrees of freedom of both throacohumeral and scapulothoracic. For each degree of freedom, the first value (black) shows the SKIN session and the second value (blue) shows the PIN session.

**Table 2.** The maximum value of SHRand its corresponding thoracohumeral elevation during SKIN and PIN sessions.

	Subject 1		Subject 2		Subject 3	
	SKIN	PIN	SKIN	PIN	SKIN	PIN
	Maximum SHR (corresponding thoracohumeral elevation °)					
Abduction	2.7 ± 0.2 (140.9 ± 31.9°)	2.7 ± 0.1 (65.3 ± 28.2°)	3.8 ± 0.3 (99.6 ± 35.2°)	3.8 ± 0.5 (82.2 ± 11.9°)	12.4 ± 1.6 (98.6 ± 5.0°)	2.9 ± 0.0 (98.5 ± 13.6°)
Adduction	2.6 ± 0.1 (149.5 ± 2.5°)	2.4 ± 0.1 (126.4 ± 23.9°)	4.1 ± 0.4 (69.7 ± 8.5°)	3.6 ± 0.2 (86.3 ± 18.3°)	14.1 ± 1.2 (95.3 ± 3.8°)	3.0 ± 0.0 (108.3 ± 0.2°)
Flexion	2.5 ± 0.0 (132.7 ± 6.8°)	2.6 ± 0.1 (140.5 ± 2.8°)	3.9 ± 0.6 (59.4 ± 22.8°)	3.2 ± 0.2 (125.9 ± 6.4°)	4.8 ± 1.1 (99.1 ± 10.9°)	4.2 ± 0.4 (38.8 ± 8.6°)
Extension	2.5 ± 0.0 (134.6 ± 6.2°)	2.6 ± 0.1 (141.1 ± 3.3°)	5.9 ± 1.6 (44.4 ± 17.3°)	3.3 ± 0.2 (113.4 ± 32.5°)	4.8 ± 1.3 (100.8 ± 12.9°)	3.6 ± 0.1 (78.4 ± 39.0°)

Abduction: elevation in frontal plane; adduction: depression in frontal plane; flexion: elevation in sagittal plane; extension: depression in sagittal plane.

All the values are reported as mean ± SD of all available trials for each task.

SKIN: session before bone-pin insertion; PIN: session after bone-pin insertion; SHR: scapulohumeral rhythm; SD: standard deviation.

*S2*: In *frontal plane*, lower thoracohumeral elevation was observed in PIN session, which was accompanied by higher external rotation and lower RoM in the plane of elevation. scapulothoracic also reduced the RoM for lateral rotation in this session (Figure 1, Table 1). Similar trend was observed for SHR during both elevation and depression for the two sessions (Figure 2). In *sagittal plane*, the elevation of thoracohumeral lowered, and the subject generated lower RoM for axial rotation. Regarding scapulothoracic kinematics, the posterior tilting increased, while the lateral rotation generated lower RoM in PIN session. In addition, SHR was higher in SKIN session than PIN session. The pattern of change in SHR showed some differences: SKIN session started with an increasing rate for SHR and started to decrease with a lower rate in the middle of elevation range, whereas the PIN session conserved the increasing rate for SHR during the whole elevation range (Figure 2, Table 2). For both sessions, S2 showed lower SHR for elevation compared to depression (Figure 2).

*S3*: In *frontal plane*, scapulothoracic kinematics handled lower posterior tilt, higher lateral rotation and lower retraction for PIN session. While the RoM for thoracohumeral elevation altered slightly, the scapulothoracic increased its RoM in all three planes of rotation after inserting pins (Figure 1, Table 1). Dramatically lower SHR was also observed in PIN compared to SKIN session (Figure 2). The pattern of SHR did not either follow similar patterns between two sessions. SHR was lower during elevation compared to depression. In both sessions maximum SHR occurred at the highest values of thoracohumeral elevation (Table 2). In *sagittal plan*e, the subject increased thoracohumeral elevation, scapulothoracic lateral rotation as well as its RoM, and scapulothoracic retraction during PIN session (Figure 1, Table 1). The different pattern for SHR was also observed for elevation/depression. An approximately constant rate in SHR was handled for elevation/depression in SKIN session. However, an increase in SHR with thoracohumeral elevation during early arm elevation was followed by a decrease at higher elevations in PIN session. During depression, the subject kept an approximately constant SHR near to the end of depression followed by decreasing rate for lower thoracohumeral elevations (Figure 2, Table 2).

### Statistical parametric mapping

**Figure 3.**
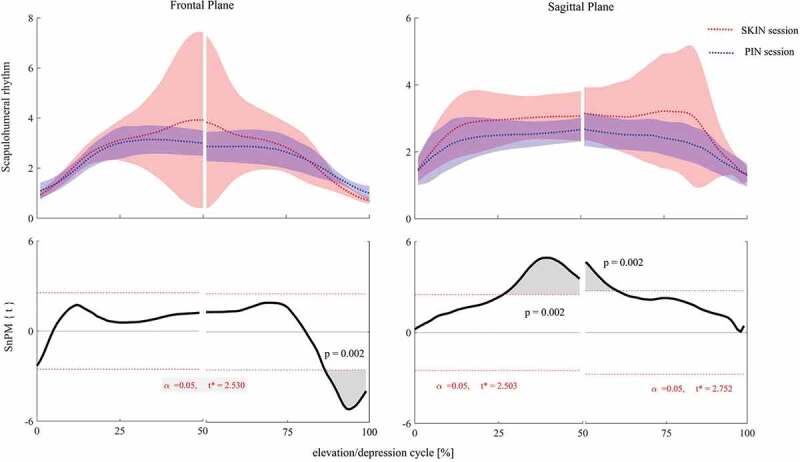
Non-parametric paired *t*-test analysis for comparing SHRduring arm elevation/depression in frontal (left) and sagittal (right) plane. Red shows SKIN session and blue shows PIN session. Thick lines show the mean value and the shaded areas show standard deviation. SKIN/PIN: session before/after bone-pin insertion.

The group mean and standard deviation plots showed some significant differences in SHR between SKIN and PIN sessions. In frontal plane, higher SHR was observed in PIN session at the end of depression phase, over 87–100% of elevation/depression phase, i.e. from 108° to 95.3° (*p =* 0.002, Figure 3). In sagittal plane, lower SHR was achieved in PIN session compared to SKIN session. However, lower SHR was just significant before and after maximum elevation over 25–61% of thoracohumeral elevation/depression, i.e. from 57° to 100° during elevation and 100° to 91.4° during depression phase (*p* = 0.002, Figure 3).

## Discussion

This study aimed to examine any changes in shoulder joint rotations and coordination as a consequence of inserting intracortical bone pins during arm elevation/depression. The results provide some insights on how bone pins may affect thoracohumeral and scapulothoracic kinematics as well as SHR. After inserting shoulder bone pins, thoracohumeral elevation and internal rotation as well as their RoMs changed during abduction, especially close to the maximum arm elevation. More obvious differences between PIN and SKIN sessions were observed for scapulothoracic kinematics during the whole cycle of elevation/depression in both frontal and sagittal plane. In addition, SHR also decreased in PIN session, i.e. less contribution of thoracohumeral joint and/or more contribution of scapulothoracic joint to arm elevation based on our kinematics results. However, SHR changes did not follow a systematic pattern across our participants. While S1 and S2 could partially produce similar SHR during SKIN and PIN sessions, S3 failed to generate similar values or patterns between sessions. S3 did not feel comfortable in PIN session, and exhibited higher scapulothoracic rotations and RoMs, specifically higher lateral rotation, after inserting pins. Due to kinematic redundancy, central nervous system might have used different strategies to execute the motion. This might explain different proportions of joint rotations after inserting pins for this subject. Based on our observation, it is suggested that future clinical studies, that are going to consider the kinematics from bone pins as gold standard, firstly identify and exclude the subjects who exhibit obviously different biomechanical outcomes after inserting pins from further statistical analysis. Although previous studies confirmed the validity of foot dynamics calculated from intracortical bone pins comprising local anaesthesia (Arndt et al. 2007; Maiwald et al. 2017), we could not completely support this assumption for shoulder coordination due to inter-subject variability. Here, the inter-subject variability might be referred to different sensitivity of subjects to the anaesthesia and psychological effect of holding pins during arm movements.

For all subjects, our results showed differences in scapulothoracic kinematics (mediolateral rotation, anteroposterior tilting and pro/retraction), while thoracohumeral kinematics just altered slightly. In fact, the subjects reduced their axial rotation and the plane of elevation after inserting pins. These alterations are consistent with findings of previous studies about shoulder abnormalities. Mell et al. (2005) showed an increase in SHR due to higher scapular motion for similar amount of humeral elevation in subjects with rotator cuff pathology. McQuade and Smidt (1998) found that SHR might change as the effect of fatigue. Higher mediolateral scapular rotation has also been mentioned to increase SHR in subjects with frozen shoulder (Vermeulen et al. 2002; Lin et al. 2006). Therefore, it can be inferred that inserting pins tend to alter scapulothoracic kinematics and SHR similar to musculoskeletal disorders.

The changes in joint kinematics before and after pin insertion (Figure 1, Table 1) were much higher than standard errors in measurement reported as [1.4° to 2.5°] for scapulohumeral kinematics and [0.09° to 4.63°] for thoracohumeral kinematics during arm elevation/depression in frontal and sagittal plane (Gonçalves et al. 2017). In order to reduce the effect of skin motion artefact in the kinematic results for both session, an advanced multibody kinematic model was used in this study (Michaud et al. 2017) which could decrease the error of thoracohumeral axial rotation to 5°, and scapula mis-orientation to 14.9° compared to scapula palpatory as well as 1.1°<root-mean-square error<3.3° for scapula kinematics compared to bone pins during arm flexion and abduction.

In addition to joint rotations, SHR was selected to look at the effect of inserting bone pins, since it can represent the movement quality index for shoulder complex for several reasons. The most important reason is that kinematic redundancy enables the central nervous system to generate a specific shoulder motion with different contribution of bony structures (Yang et al. 2007; Fayad et al. 2008). Patients with shoulder abnormalities could alter the contribution of shoulder joints to provide stability and proper RoM thanks to such redundancy (Fayad et al. 2008; Braman et al. 2009; Forte et al. 2009). A further point is that SHR can be more robust than individual shoulder joint kinematics due to involving less between-subject variability (McQuade and Smidt 1998). Additionally, in case of our study, the participants were not instructed to perform maximum RoM either before or after pin insertion; the same experimenter was however in front of each participant to show each movement. This limits the comparison above 108° of arm elevation. Due to the small sample size (*n* = 3) in our cross-comparison study, we compared the kinematics of each subject separately between SKIN and PIN sessions. In addition, the obvious differences in SHR between subjects might be due to individual movement strategies rather than different compensatory mechanisms. It is recommended that validation studies which mount both skin markers and bone-pin markers on the body estimate how they will interact with each other before data collection. Otherwise, repositioning skin markers because of interference with bone pins would deteriorate the kinematic results. In our study, one of our four available subjects was excluded due to this issue. A well-organized protocol especially for such invasive studies with small sample sizes will help in minimizing the between-subject differences derived from methodological errors. While only kinematics were assessed in the present study, electromyography and/or muscle force estimation may provide additional information about the effect of pin insertion on the upper-limb biomechanics. However, the estimation of muscle forces at the shoulder remains challenging due to co-contraction and the complex trajectories of (multi-articular) muscles (Behm et al. 2003; De Sapio et al. 2006). Some advanced algorithms are developed based on the tracking of both EMG and kinematics which accounts for the excitation pattern of each individual (Pizzolato et al. 2015; Bélaise et al. 2018). The effect of bone pins on muscle activations could then be compared to the effect of soft tissue artefact as quantified by (Blache and Begon 2018). Bone-pin insertion and local anaesthesia might alter the complementary action of scapulothoracic and glenohumeral muscles to produce the complex kinematics and rhythm during arm elevation/depression (Yoshizaki et al. 2009). Therefore, adding muscle activation would improve the clinical benefits of such invasive studies.

## Conclusion

Our results showed that inserting shoulder bone pins dominantly deteriorates the pattern and RoM of scapulothoracic joint rather than thoracohumeral joint. It was also observed that SHRmight be partially reproduced after pin insertion. Eliminating methodological errors from soft tissue artefacts using intracortical pins would not necessarily add more clinical value to the kinematic results, while the approach remains relevant to model/algorithm validation.
